# Chronic cystoisosporiasis in an immunocompetent adult: A case report

**DOI:** 10.1097/MD.0000000000024890

**Published:** 2021-03-12

**Authors:** Masashi Ohno, Osamu Inatomi, Takayuki Imai, Kenichiro Takahashi, Shigeki Bamba, Keiji Konishi, Masaya Sasaki, Ryoji Kushima, Akira Andoh

**Affiliations:** a Department of Medicine; b Division of Digestive Endoscopy; c Division of Clinical Nutrition; d Department of Pathology, Shiga University of Medical Science; e Department of Infectious Disease, Osaka City General Hospital, Japan.

**Keywords:** capsule endoscopy, case report, *cystoisospora belli*, cystoisosporiasis, immunocompetent

## Abstract

**Rationale:**

Cystoisosporiasis is an intestinal infectious disease caused by a coccidian protozoa, *Cystoisospora belli* (*C. belli*). It can cause prolonged and refractory diarrhea most commonly in immunocompromised patients, while immunocompetent individuals usually exhibit no symptoms or self-limited diarrhea.

**Patient concerns:**

We herein report a case of chronic cystoisosporiasis in an immunocompetent patient. A 62-year-old man, who had been first diagnosed with cystoisosporiasis 15 years ago and had been treated with oral administration of trimethoprim-sulfamethoxazole (TMP-SMX), complained of persistent watery diarrhea. He was negative for anti-human immunodeficiency virus antibody and anti-human T-cell leukemia virus type 1 (HTLV-1) antibody.

**Diagnosis:**

Biopsy specimens from the duodenum revealed oocysts in the atrophic absorptive epithelium and protozoa were detected through stool examination, indicating the recurrence of cystoisosporiasis. Capsule endoscopy showed diffuse atrophic mucosa with white villi in the entire small intestine. We diagnosed him with chronic cystoisosporiasis that occurred in an immunocompetent adult.

**Interventions:**

Since oral administration of TMP-SMX and ciprofloxacin were ineffective, the intravenous administration of TMP-SMX was initiated.

**Outcomes:**

Intravenous TMP-SMX exhibited a significant improvement.

**Lessons:**

This case indicates that even immunocompetent individuals may develop recurrent and refractory cystoisosporiasis. Furthermore, intravenous treatment of antibiotic agents should be considered when the impaired absorptive ability from the small intestine is suspected.

## 1 Introduction

*Cystoisospora belli* (*C. belli*) is a protozoan parasite that primarily infects the epithelial cells of the small intestine.^[1]^ It is present worldwide but is mostly found in tropics and subtropics.^[1]^ Humans are thought to be only a host of *C. belli* since there is no report that *C. belli* infects other animals.^[2]^ People become infected by ingesting oocysts excreted from the stool of infected patients.^[3]^*C. belli* infection, called cystoisosporiasis, is characterized by watery and non-bloody diarrhea. Most cases are reported in patients who are immunocompromised hosts with acquired immune deficiency syndrome (AIDS) or adult T-cell leukemia (ATL).^[4,5]^ Diarrhea is usually prolonged and refractory and leads to malabsorption syndrome.^[6]^ In contrast, *C. belli* infection is often either asymptomatic or associated with self-limited acute diarrhea in immunocompetent hosts.^[6]^

According to the therapeutic guidelines for patients with AIDS, trimethoprim-sulfamethoxazole (TMP-SMX) is recommended as the first-line antibiotic agent for cystoisosporiasis.^[5]^ When TMP-SMX is ineffective or intolerant, ciprofloxacin should be considered as an alternative treatment.^[5,7]^ However, in cases of severe damage to epithelial cells of the small intestine, these medicines are not absorbed adequately. In this situation, it is recommended to administer medicines intravenously.^[5]^ Here, we report a case of chronic *C. belli* infection that occurred in an immunocompetent adult. Capsule endoscopy and histopathological findings revealed the severe atrophic mucosa in the small intestine. The patient was successfully treated by intravenous administration of TMP-SMX, but not its oral administration.

## 2 Case report

**Figure 1:**
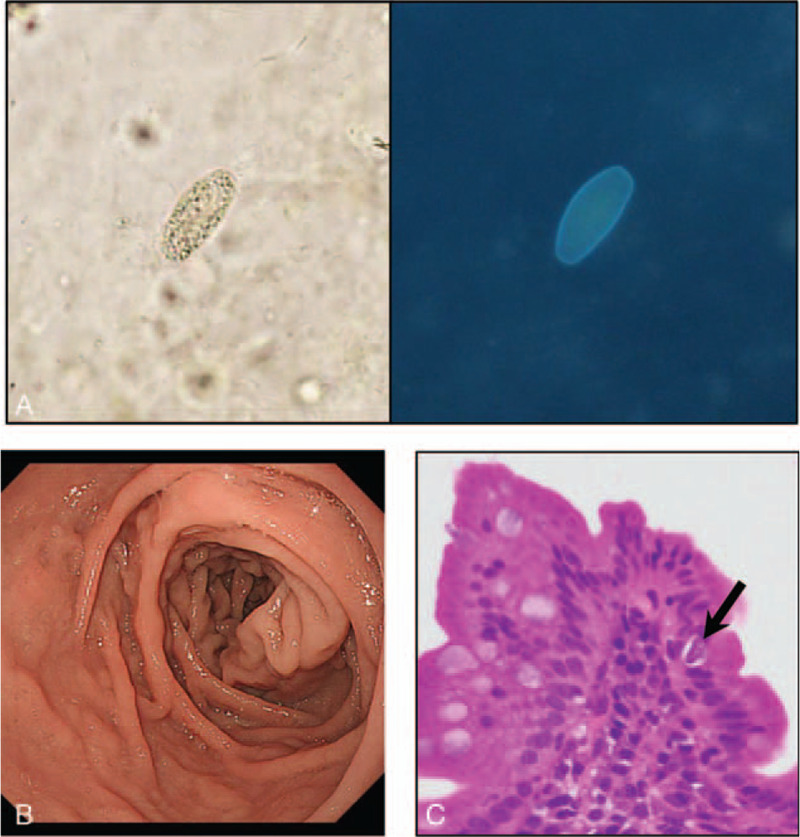
(A) A light microscopic image (left panel) and UV fluorescence microscopy image (right panel) of the stool. (B) EGD showed the marked atrophic mucosa in the duodenum. (C) The H/E-stained section revealed the presence of an oocyst within the absorptive epithelial cells (solid arrow). Original magnification × 100.

**Figure 2:**
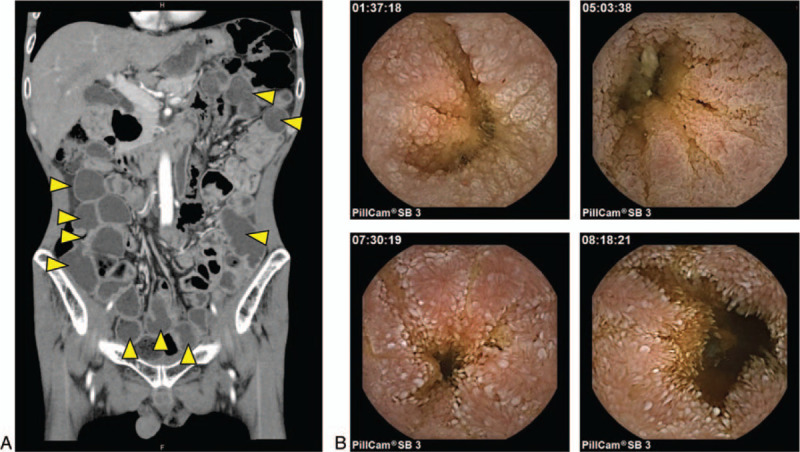
(A) Contrast-enhanced CT on admission. The small intestine contained excessive fluid (Yellow Arrowheads) but showed no obvious wall thickness. (B-E) Capsule endoscopy showed unique images with white villi in the entire small intestine. Small bowel transit time was 8 hour 21 minutes.

A 62-year-old man visited the out-patient department of the Shiga University of Medical Science Hospital with prolonged watery diarrhea and lower-extremity weakness. He was once diagnosed with *Cystoisospora* infection 15 years ago and treated with oral TMP-SMX. However, diarrhea lasted intermittently after the treatment. Laboratory studies on visit showed eosinophilia (778 /μl), hypoalbuminemia (3.3 g/dl), hypokalemia (3.0 mmol/L), and normal serum C-reactive protein (0.02 mg/dl) (Table 1). He was born and raised on a small island located in south Kyushu, Japan, where human T-cell leukemia virus type 1 (HTLV-1) is endemic, and his father died with ATL. However, he was negative for anti-HTLV-1 antibody (Table 1). Additional laboratory examinations such as human immunodeficiency virus antigen/antibody test, serum soluble interleukin-2 receptor, the CD4 count were normal (Table 1). We performed a stool microscopy examination and found the presence of protozoa that exhibited green autofluorescence under ultraviolet exposure (Fig. 1A). Consistently, esophagogastroduodenoscopy showed diffuse atrophic mucosa in the duodenum (Fig. 1B) and a microscopic examination of the duodenum biopsy specimens revealed invaded oocysts in the atrophic absorptive epithelial cells (Fig. 1C). A contrast-enhanced computed tomography indicated excess fluid in the entire small intestine with no wall thickness (Fig. 2A). Therefore, he underwent capsule endoscopy (PillCam SB 3; Medtronic, Dublin, Ireland) to observe the mucosa of the small intestine and found the atrophic mucosa with diffuse white villi in the entire small intestine and no apparent erosions and ulcers (Fig. 2B). These findings indicated chronic infection with *C. belli*.

**Table 1:** Laboratory findings on admission.

Parameter	Result	Normal Range	Parameter	Result	Normal Range
WBC (/μl)	6600	3000–8000	CRP (mg/dl)	0.02	000–0.30
Neut (%)	62.3	40.0–74.0	IgM (mg/dl)	50	35–220
Lymph (%)	18.8	15.0–48.0	IgG (mg/dl)	1312	870–1700
Eosin (%)	11.8	0.0–7.0	IgA (mg/dl)	412	110–410
Mono (%)	6.2	2.0–12.0	IgE (IU/ml)	3901.1	0.0–400.0
CD4 (/μl)	870	500–1200	sIL-2R (U/ml)	418	121–613
Hb (g/dl)	13.1	12.4–17.0	Glucose (mg/dl)	108	70–109
Ht (%)	36.5	38.0–54.0	HbA1c (%)	5.4	4.6–6.2
Plt (x10^4^/μl)	21.0	15.0–40.0	ESR (mm/hour)	5.0	2.0–10.0
TP (g/dl)	6.3	6.3–8.3	ANA	(−)	
Alb (g/dL)	3.3	4.0–5.0	HBs-Ag	(−)	
Na (mEq/L)	139	138–146	HCV-Ab	(−)	
K (mEq/L)	3.0	3.6–4.9	HIV-Ag/Ab	(−)	
Cl (mEq/L)	109	99–109	TP-Ag	(−)	
T. chol (mg/dl)	127	128–219	HTLV1-Ab	(−)	
TG (mg/dl)	57	30–149			

Alb = albumin, ANA = anti-nuclear antibody, CRP = C-reactive protein, Eosin = eosinophil, ESR = erythrocyte sedimentation rate, Hb = hemoglobin, HbA1c = glycated hemoglobin, HBs-Ag = hepatitis B surface antigen, HCV-Ab = hepatitis C virus antibody, HIV-Ag/Ab = human immunodeficiency virus antigen/antibody, Ht = hematocrit, HTLV-1-Ab = human T cell leukemia virus type 1 antibody, Lymph = lymphocyte, Mono = monocyte, Neut = neutrophil, Plt = platelet, sIL-2R = soluble interleukin-2 receptor, T. chol = total cholesterol, TG = triglyceride, TP = total protein, TP-Ag = treponema pallidum antigen, WBC = white blood cell.

We started oral administration of 2 tablets of trimethoprim-sulfamethoxazole (TMP-SMX; 160 mg of TMP, 800 mg of SMX per day), followed by ciprofloxacin (400 mg per day). However, both failed to improve the patient's symptoms. Afterward, he received central parenteral nutrition and was treated with intravenous administration of TMP-SMX (15 mg per kg per day based on TMP component) for 10 days, since an impairment of drug absorption was suspected. After this, his symptoms were remarkably improved, and the protozoa became undetectable in the stool. So far, 9 months have passed since the intravenous intervention, and there is no evidence of recurrence.

## 3 Discussion

We experienced a case of chronic *C. belli* infection in an immunocompetent adult. *C. belli* is a protozoan pathogen that causes an opportunistic infection primarily in immunocompromised patients with AIDS and/or ATL.^[8]^ Cystoisosporiasis is also reported in patients with non-Hodgkin lymphoma, alcoholism, and ulcerative colitis taking azathioprine and anti-TNF-α antibody treatment.^[9–11]^ However, only a few reports described *C. belli* infection in immunocompetent individuals.^[12,13]^ In our case, there was no evidence of immunocompromised status by extensive examinations. Given that ATL was endemic in his birthplace, it is speculated that he might be infected with *C. belli* in infancy and become chronic. Furthermore, *C. belli* infection was chronic because the patient exhibited intermittent diarrhea for more than 15 years. Severe atrophic mucosa observed in capsule endoscopy also supported chronic infection. These endoscopic images are invaluable in understanding how cystoisosporiasis affects the small intestinal mucosa.

The diagnosis of *C. belli* infection is usually based on the detection of oocysts in the stool. It can be also found in an intestinal biopsy specimen.^[5]^ However, it can be difficult to detect by routine examinations. Therefore, the detection of autofluorescence is helpful to identify *C. belli* in the stool when *C. belli* infection is suspected.^[14]^ Interestingly, our case showed prominent eosinophilia that is one of the unique features of cystoisosporiasis.^[15]^ Other protozoan infections generally do not cause eosinophilia; this finding is useful for diagnosing cystoisosporiasis. Of note, our case also showed markedly elevated serum IgE levels, suggesting an excess allergic response in the small intestine.

Treatment of cystoisosporiasis was usually performed with oral administration of antibiotic agents. However, the oral administration of drugs was ineffective in our patients. This is presumably due to the impaired absorption ability associating with severe mucosal atrophy. It should be noted that intravenous administration of antibiotic agents is recommended for the treatment of *C. belli* infection when impaired mucosal absorption is suspected.^[5]^ Indeed, intravenously administered TMP-SMX quickly improved his symptoms.

In conclusion, this case emphasizes that immunocompetent individuals can develop chronic and recurrent cystoisosporiasis. Cystoisosporiasis should be taken into account in patients with chronic diarrhea who have mucosal atrophy in the small intestine. In addition, capsule endoscopy is thought to be a useful tool for the early detection of mucosal atrophy.

## Author contributions

**Conceptualization:** Masashi Ohno, Osamu Inatomi.

**Funding acquisition:** Masashi Ohno.

**Investigation:** Masashi Ohno, Takayuki Imai, Kenichiro Takahashi, Keiji Konishi, Ryoji Kushima.

**Supervision:** Osamu Inatomi, Akira Andoh.

**Validation:** Shigeki Bamba, Masaya Sasaki.

**Writing – original draft:** Masashi Ohno.
